# Care of Patients After Bariatric Surgery in the Periconceptional and Perinatal Periods

**DOI:** 10.3390/nu18081280

**Published:** 2026-04-17

**Authors:** Karolina Skulimowska, Tomasz Tomkalski, Agata Góral, Marek Murawski

**Affiliations:** 1 Department of Endocrinology, Diabetology and Internal Medicine, Lower Silesian Specialist Hospital-Emergency Medicine Center, 54-049 Wroclaw, Poland; 2 Faculty of Medicine, Wroclaw University of Science and Technology, 50-386 Wroclaw, Poland; 3 University Clinical Hospital, 50-556 Wroclaw, Poland; 4 Clinical Department of Gynecological Surgery and Oncology, Wroclaw Medical University, 50-556 Wroclaw, Poland

**Keywords:** bariatric surgery, contraception, pregnancy, delivery, postpartum period

## Abstract

Obesity in women of reproductive age is a major issue. It is associated with reduced fertility and an increased risk of obstetric and perinatal complications. Bariatric surgery is the most effective treatment for severe obesity, leading to substantial weight reduction, improvement of metabolic disorders, and enhanced fertility. Consequently, an increasing number of women are becoming pregnant after undergoing bariatric surgery. The aim of this paper is to review current recommendations and research data regarding the care of women after bariatric surgery in the periconceptional and perinatal periods, as well as throughout pregnancy, delivery, and the postpartum period. Research suggests that pregnancy after bariatric surgery is associated with a lower risk of gestational diabetes, hypertension, preeclampsia, and fetal macrosomia compared with pregnancies in women with similar baseline BMI (body mass index) who have not undergone surgical treatment. At the same time, an increased risk is observed for low birth weight, maternal micro- and macronutrient deficiencies, and complications characteristic of bariatric procedures, such as dumping syndrome or intra-abdominal hernias. Most scientific societies recommend postponing pregnancy planning for 12–18 months after surgery and using effective contraception, preferably methods that do not require gastrointestinal absorption. Regular monitoring of laboratory parameters, individually tailored supplementation, and interdisciplinary care are essential for the safe management of pregnancy after bariatric surgery. In particular, care should include achieving a stable body weight before conception, monitoring of nutritional status, verifying proper weight gain during pregnancy, and considering alternative methods for gestational diabetes screening (e.g., glycaemic monitoring instead of oral glucose tolerance testing) due to the risk of dumping syndrome. Appropriate preparation for pregnancy and proper management throughout its course allow for reducing the risk of perinatal complications. Bariatric surgery itself is not a contraindication to vaginal delivery.

## 1. Introduction

The global prevalence of obesity is steadily increasing. According to WHO data from 2022, 2.5 billion adults have excess body weight, with over 890 million people living with obesity. In women, this represents about 44% [1]. This issue is becoming increasingly common among women of reproductive age. In the United States, the number of women with obesity has risen in recent years, reaching 16% in those aged 12–19 and 29% in those aged 20–39 [2].

Excess body weight not only increases the risk of type 2 diabetes, high blood pressure, cardiovascular complications, and selected cancers, but also leads to fertility issues [3,4]. Women with obesity may develop polycystic ovary syndrome, which is characterized by insulin resistance, decreased SHBG (sex hormone-binding globulin), elevated androgen concentrations, higher levels of free estrogen, and an increased LH (luteinizing hormone)-to-FSH (follicle-stimulating hormone) ratio. These changes can cause menstrual irregularities, including anovulatory cycles, and ultimately decrease fertility. Furthermore, increased leptin concentrations in women with excess body weight disrupt folliculogenesis [3,5]. Women with obesity are more likely to experience sexual dysfunction than women with a normal body weight, which may also contribute to difficulties in conceiving [6].

Pregnancy in women with obesity is also associated with an increased risk of complications for both the mother and the child. In pregnant women with obesity, there is a higher incidence of miscarriage, as well as an increased risk of developing gestational diabetes, hypertension, preeclampsia, obstructive sleep apnea, and thromboembolic complications. Furthermore, there is an increased risk of epidural anesthesia failure, peripartum injury, and postpartum hemorrhage, along with a higher rate of operative deliveries and an increased need for cesarean section [7,8,9,10]. The infants tend to have a higher birth weight, and the risk of shoulder dystocia is elevated. There is also a greater likelihood of congenital disorders such as neural tube defects, cardiac defects, cleft lip and palate, hydrocephalus, hypospadias, anal atresia, and renal cystic disease [11]. Therefore, an increasing number of women of reproductive age are choosing bariatric surgery as an effective method of reducing excess body weight [12].

Compared with other methods of obesity treatment, bariatric procedures remain the most effective therapeutic option for individuals with the most severe forms of obesity. However, it is important to remember that bariatric surgery is part of a multidisciplinary treatment approach that also involves nutritional counseling, psychological support, pharmacological therapy, and consultations with specialists managing obesity-related complications. Indications for surgical treatment include class III obesity (BMI ≥ 40 kg/m^2^) or class II obesity (BMI 35–39.9 kg/m^2^) when ≥1 major obesity-related complication is present, such as type 2 diabetes, hypertension, hyperlipidemia, or fatty liver disease [13].

There are three main types of bariatric surgery: **(1) restrictive procedures**: SG—sleeve gastrectomy, and LAGB—laparoscopic adjustable gastric banding, which limit the volume of the stomach and thus the volume of food intake; **(2) malabsorptive procedures:** BPD—biliopancreatic diversion, which bypasses part of the small intestine, decreasing the surface available for nutrient absorption; and **(3) combined restrictive/malabsorptive procedures:** RYGB—Roux-en-Y gastric bypass, MGB = OAGB − mini gastric bypass = one anastomosis gastric bypass, which combine gastric volume reduction with bypassing part of the gastrointestinal tract.

According to global statistics from 2024, the most commonly performed bariatric procedure is SG, followed by RYGB and OAGB (MGB) [14].

The aim of this paper is to review current recommendations and research regarding the care of women in the periconceptional and perinatal periods with particular emphasis on nutrition, maternal fetal outcomes and potential complications, following bariatric surgery.

## 2. Materials and Methods

Due to the broad scope of the current review, encompassing multiple clinical domains including nutritional management, obstetric outcomes, and periconception care, a narrative rather than systematic review was deemed most appropriate. The heterogeneity of available study designs and outcome measures further precluded a meaningful quantitative synthesis. Nevertheless, a structured and reproducible literature search was carried out. A comprehensive search of MEDLINE (via PubMed) and Google Scholar was conducted. The last search was carried out on 1 February 2026. Screening was performed by the authors, with discrepancies resolved through discussion and consensus.

The inclusion criteria were as follows:

- Guidelines and consensus statements issued by national and international scientific societies in the fields of metabolic bariatric surgery, endocrinology, gynecology, and perinatology;
- Original studies evaluating the impact of metabolic bariatric surgery on pregnancy and associated maternal and fetal outcomes;
- Original studies addressing macro- and micronutrient deficiencies following bariatric surgery;
- Systematic reviews and meta-analyses related to the above topics;
- Case reports or case series related to the above topics;
- Articles published in English.

The exclusion criteria were as follows:

- Articles published in languages other than English or Polish;
- Articles not accessible in full text.

The search strategy was based on combinations of the following keywords: “bariatric surgery”, “metabolic bariatric surgery”, “nutritional deficiencies”, “pregnancy”, “maternal outcomes”, “fetal outcomes”, “supplementation”, “fertility”, “reproductive health”, “lactation”, “postpartum” and “contraception”, combined using Boolean operators as appropriate. A representative search string used in PubMed was as follows: (“bariatric surgery” OR “metabolic bariatric surgery”) AND (“pregnancy” OR “maternal outcomes” OR “fetal outcomes” OR “reproductive health”).

Titles and abstracts were screened for relevance, followed by full-text assessment of eligible articles.

Greater emphasis was placed on higher levels of evidence, including guidelines, systematic reviews, and meta-analyses, while individual studies and case reports were used as complementary evidence.

The search identified the following categories of publications: 29 original research articles, 12 systematic reviews or meta-analyses, 27 narrative reviews and editorials, 13 guidelines or consensus statements, 2 case reports, and 2 book chapters.

## 3. Fertility and Reproductive Health After Bariatric Surgery

Many patients who experienced difficulty conceiving before surgery are able to achieve pregnancy afterward. The extent of weight loss plays the most significant role in improving fertility. The lowest body weight after bariatric surgery is typically reached between 12 and 18 months postoperatively. Afterward, a predictable degree of weight regain may occur [15]. Menstrual cycles become more regular, which is often associated with remission of pre-existing PCOS and normalization of hormone levels. Women also experience improvements in sexual function following the procedure [12,16,17]. It should be noted that pregnancy within the first several months after surgery is discouraged by most authors: for 12–18 months according to the recommendations of the American Society for Metabolic and Bariatric Surgery, and for 12–24 months according to the American College of Obstetricians and Gynecologists [18,19]. This is due to the intense weight loss and nutritional deficiencies that typically occur during this time, which may increase the risk of the fetus being small for gestational age (SGA). SGA is more commonly seen in pregnancies conceived within the first 6 months and between 6 and 12 months postoperatively than in those occurring 2–5 years after surgery [20]. Other studies also demonstrate that pregnancies conceived less than one year after surgery are associated with a higher risk of miscarriage [15,21]. However, the recommendations regarding the interval between bariatric surgery and pregnancy are not supported by strong scientific evidence. Given the different data in the guidelines, a consensus on the optimal pregnancy time in this group of patients should be based on the clinical presentation, patient’s age, fertility potential and reproductive plans of the patient. However, potential pregnancy complications should be kept in mind.

## 4. Contraception After Bariatric Surgery

The rapid weight loss following bariatric surgery is associated with an increased risk of unplanned pregnancy. This is particularly relevant for women who previously experienced anovulation due to polycystic ovary syndrome (PCOS), as they are more likely not to use any form of contraception before surgery [22]. Selecting an effective contraceptive method should be tailored to the individual and depend on the type of bariatric procedure performed, the patient’s medical history, and her personal preferences. Patients most frequently choose oral contraception, which has a Pearl Index of 0.3 under ideal conditions [23]. However, this index increases after bariatric procedures, partly due to changes in gastrointestinal physiology [24]. Impact of bariatric surgery on oral contraceptive effectiveness differs depending on the type of procedure. Malabsorptive and combined restrictive/malabsorptive procedures are associated with changes in the pharmacokinetics of certain medications (including estrogen, which is absorbed in the upper gastrointestinal tract that is altered during these surgeries) potentially leading to reduced absorption. Furthermore, following bariatric surgery, the pH of the gastrointestinal tract may change due to decreased gastric acid secretion. In contrast to malabsorptive and combined restrictive/malabsorptive procedures, restrictive surgeries do not cause malabsorption themselves, but some patients may experience severe vomiting or diarrhea, which can lead to impaired absorption of oral contraceptives [25,26,27]. What is also important is that some studies report reduced contraceptive effectiveness in obese women, particularly in those with a BMI > 35 kg/m^2^ who use oral contraceptive pills. Studies also indicate reduced effectiveness of contraceptive patches containing norelgestromin and ethinyl estradiol in women weighing more than 90 kg [28,29]. Moreover, women with a BMI > 30 kg/m^2^ who take combined oral contraceptive pills have a slightly higher risk of thromboembolic events than obese women who do not use oral contraceptives [30].

Therefore, it is worth considering the use of subdermal implants or levonorgestrel-releasing intrauterine devices for contraception after bariatric surgery, as these appear to be safer options compared with hormonal oral contraception, and are associated with a lower risk of failure and adverse effects [24,31].

## 5. Pregnancy After Bariatric Surgery

Gestational weight gain is a key factor in determining both maternal and fetal outcomes. To prevent complications resulting from insufficient or excessive gestational weight gain, a stable body weight should be achieved before conceiving after bariatric surgery, and weight should be monitored throughout the entire pregnancy. An important factor is reaching the recommended pregnancy weight gain determined on the basis of pre-pregnancy BMI, as outlined in the Institute of Medicine (IOM) guidelines. Physiological weight gain should occur in every pregnancy, even in individuals with obesity, as shown in Table 1 [3,32].

Recommended gestational weight gain may be difficult to achieve due to an insufficient interval between surgery and conception, reduced food intake and absorption depending on the type of procedure performed, as well as the woman’s potential fear of regaining weight [33,34].

Insufficient gestational weight gain may result in an increased risk of fetal SGA and preterm birth [35]. Studies have shown that the type of bariatric procedure (malabsorptive or combined restrictive/malabsorptive, e.g., gastric bypass) and the associated weight loss may increase the risk of lower neonatal birth weight and fetal SGA [33]. Low birth weight can predispose the child to future cardiac, metabolic, neurological, and psychiatric complications [36]. Conversely, excessive gestational weight gain may lead to LGA (large for gestational age) and a range of obstetric complications, as well as health consequences for the child, including neonatal hypoglycemia, prolonged hospitalization, and an increased risk of developing obesity in adulthood [37,38,39].

Despite the risks related to fetal grow, bariatric surgery is associated with significant maternal metabolic benefits. Compared to pregnancies in woman with the same preoperative BMI, the odds of gestational diabetes are significantly reduced (OR 0.20, 95% CI, 0.11–0.37), as are the odds of hypertension and their complications, such as preeclampsia (OR 0.38; 95% CI, 0.27–0.53) [8,40].

These outcomes may vary depending on the type of the procedure: hypertension, preeclampsia, and LGA are observed less often than in those after sleeve gastrectomy, while gestational diabetes occurs less frequently following sleeve gastrectomy [19]. There are no significant differences between bariatric surgery types in terms of the rates of miscarriage or stillbirth [7].

Pregnancy following bariatric surgery also exposes patients to the risks associated with the prior procedure. Internal hernias occur in about 5% of patients after gastric bypass procedures and may present with abdominal pain or even, in severe cases, bowel obstruction. This is mainly due to elevated intra-abdominal pressure and compression of the intestines by the gravid uterus [41]. Following gastric banding, the risk of band slippage during pregnancy is about 12% compared with about 4% outside pregnancy [42]. It is also important to consider the possibility of psychiatric disorders in these patients, with particular emphasis on anxiety [22].

Dumping syndrome is another important complication, affecting 40–76% of patients after RYGB and up to 40% of patients after LSG. The early type typically appears about 1 h after eating and presents with symptoms as diarrhea, sweating, flushing, and tachycardia. The late type is associated with hypoglycemic symptoms occurring 1–3 h after consuming a carbohydrate-containing meal, which triggers an excessive insulin response [43]. For this reason, given the risk of dumping syndrome in pregnant women after bariatric surgery, the OGTT (oral glucose tolerance test) is not recommended between 24 and 28 weeks of gestation for the diagnosis of gestational diabetes. To monitor the risk of diabetes, measurement of glycated hemoglobin (HbA1c) is recommended at the beginning of pregnancy, and a 7-day period of daily glucose monitoring (fasting and 2 h postprandial) is advised between 24- and 28-week of pregnancy [44].

Pregnancy following bariatric surgery is associated with significant benefits for both mother and fetus, as well as an increased risk of certain adverse outcomes, as shown in Table 2.

## 6. Micro- and Macronutrient Deficiencies in Pregnant Women After Bariatric Surgery

Pregnancy after bariatric surgery may also result in micro- and macronutrient deficiencies in both the mother and the fetus. Therefore, it is essential to ensure proper preconception preparation through appropriate nutritional counseling [42]. These deficiencies occur as a result of restricted food intake, reduced nutrient absorption, and the increased nutritional demands of pregnancy [45]. Additionally, they may be exacerbated by the nausea and vomiting that are common during the first trimester of pregnancy [22]. Different types of bariatric procedures may lead to different micronutrient deficiencies during pregnancy, which can result in low birth weight, congenital disorders, and other long-term consequences, as well as an increased risk of miscarriage, preterm birth, or stillbirth [46]. Regardless of the surgical technique used, it is necessary to maintain continuous supervision of the patient, monitor micro- and macronutrient levels, and provide supplementation tailored to the patient’s rising nutritional requirements during pregnancy. This will help prevent adverse consequences for both the mother and the child.

In pregnancy after bariatric surgery, the most common deficiencies include:

a. Vitamin B12

Vitamin B12 deficiency can occur after any type of bariatric surgery, but it is most commonly seen after combined restrictive/malabsorptive procedures like RYGB [26]. This is due to the reduced gastric surface area that produces intrinsic factor (IF), necessary for vitamin absorption, and to the bypass of the duodenum and proximal small intestine, which are the primary sites of micronutrient absorption [47,48]. Vitamin B12 levels should be measured as early as three months before the planned conception, and its supplementation should be started before folic acid is initiated. For prevention, it is recommended to administer 1 mg of vitamin B12 orally per day or 1000 µg intramuscularly every 12 weeks [22]. During the first two years after bariatric surgery, vitamin B12 deficiencies may not be observed because of the liver’s stored reserves [48].

b. Folic acid

Folic acid supplementation should be initiated as early as three months before the planned conception, as it significantly reduces the risk of neural tube defects, miscarriage, and congenital heart defects [49]. However, excessively high folate intake may affect the child’s psychomotor development and increase the risk of childhood asthma [50]. Currently, there are no definitive guidelines on the recommended folic acid dose for pregnant patients following bariatric surgery. The British Obesity & Metabolic Surgery Society recommends 4 to 5 mg of folic acid per day for obese women in the periconceptional period [51]. However, most scientific societies, including the American Association of Clinical Endocrinologists, the American Society for Metabolic & Bariatric Surgery, and Obesity Canada, recommend supplementation not exceeding 1 mg per day [18,51].

The National Institutes of Health recommend the use of 400 µg of folic acid per day for every woman who is planning a pregnancy and for those in the first 12 weeks of gestation with no indication for women with a BMI > 25 kg/m^2^ to take larger doses. However, it is noted that women with a history of bariatric surgery should consult their bariatric center for personalized supplementation advice [52].

c. Iron

Iron-deficiency anemia is a particularly common long-term complication after bariatric surgery, especially following malabsorptive procedures such as RYGB [53]. During this procedure, the duodenum and the proximal jejunum are bypassed, which reduces the surface area where iron absorption is most intensive. Some studies also report that RYGB leads to a decrease in villus height, which additionally hinders iron absorption. Bariatric surgery also results in decreased secretion of hydrochloric acid, whose role is to convert iron from the ferric to the ferrous form, which is more readily absorbed by the body [54]. Research comparing iron levels before and after bariatric surgery demonstrates a marked reduction in iron levels postoperatively [55]. During pregnancy, the demand for iron grows due to the increased blood volume. In pregnant women, anemia is diagnosed when hemoglobin levels fall below 11 g/dL in any of the three trimesters [56]. Symptoms indicative of anemia include increased fatigue, pallor, dizziness, and weakness. Iron deficiency in the mother, especially during the first trimester, negatively affects the fetus and may result in fetal growth restriction, preterm birth, and low birth weight [57]. Guidelines for supplementation after bariatric surgery recommend 45–60 mg of elemental iron [48,58,59].

d. Fat-soluble vitamins—A, D, E, and K

Vitamin A deficiency occurs in nearly 90% of post-bariatric pregnancies. It is associated with inadequate maternal weight gain, and may lead to visual impairment, fetal growth restriction, and preterm birth [60,61]. For vitamin A, supplementation should be provided in the form of beta-carotene rather than retinol, which may have teratogenic effects, particularly in the first trimester of pregnancy. Therefore, if a patient has been taking supplements containing vitamin A prior to pregnancy, it may be necessary to switch to a product that does not contain retinol. The daily dose of vitamin A should not exceed 5000 IU per day [62].

The standard recommended supplementation of 25-OH-D3 is 1000 IU per day. Higher doses are advised in cases of deficiency, depending on its severity, and may reach 3000–6000 IU per day. The concentration of 25-OH-D3 should be maintained at 50 nmol/L [22]. In cases of deficiency, additional calcium supplementation of 1000–2000 mg per day is also recommended [63]. Vitamin D and calcium deficiencies may lead to a secondary increase in parathyroid hormone (PTH) secretion [64].

Vitamin K and E deficiencies can develop after malabsorptive and combined restrictive/malabsorptive procedures. A 2023 meta-analysis demonstrated an association between reduced vitamin E levels in pregnant women and a higher risk of adverse pregnancy outcomes, specifically preeclampsia, miscarriage, and low birth weight [65]. This relationship is further confirmed by another study showing a higher risk of preeclampsia among women with vitamin E levels below 7.3 mg/L during the first trimester [66]. Vitamin K is essential for the synthesis of prothrombin and other coagulation factors. Therefore, its deficiency leads to hemorrhagic complications in both the mother and the child [67,68]. Regarding neonatal complications, there have been several reports of intracranial hemorrhage in infants resulting from maternal vitamin K deficiency [69,70]. Vitamin K does not cross the placenta efficiently. Therefore, newborns are born with low vitamin K levels, even when maternal levels are normal [67]. The recommended dose of vitamin E is 15 mg, whereas in cases of confirmed or suspected vitamin K deficiency (indicated by coagulation disorders) a weekly oral dose of 10 mg is advised [22,71]. To prevent severe hemorrhagic complications, all newborns should be administered 1 mg of vitamin K intramuscularly within the first 24 h after birth [72].

e. B vitamins

Women experiencing nausea and vomiting during pregnancy, as well as those with significant weight loss, are particularly susceptible to deficiencies in B vitamins, especially vitamin B1. The latter may lead to beriberi or Wernicke’s encephalopathy. It is generally recommended to supplement thiamine at a dose of 12 mg per day. For women experiencing vomiting, thiamine at 300 mg in a B-complex formulation, taken three times a day, is often advised. When oral administration is not possible, a dose of 100 mg should be administered intramuscularly [22,37,73].

f. Protein

Protein deficiency is one of the most common macronutrient deficiencies following bariatric surgery. It is most frequently seen after BPD and RYGB due to malabsorption and impaired digestion. LSG and LAGB may also lead to protein deficiency if dietary preferences change and persistent vomiting occurs [74,75,76]. Daily protein intake should reach 60–120 g per day, ideally 1.5 g/kg of body weight per day. It should also be emphasized that protein intake following BPD needs to be increased by 30%. If dietary protein intake is insufficient, liquid protein supplementation should be introduced. Protein deficiency is indicated by an albumin concentration below 3.5 g/dL, together with clinical symptoms like hair loss, muscle loss, and edema [74,76].

g. Selenium

Some studies suggest that selenium deficiency may contribute to miscarriage, whereas excessive intake may lead to neurological damage. The recommended supplementation dose is 50 µg per day [22].

h. Zinc and Copper

For zinc and copper, research data are inconclusive, and their deficiencies in pregnant women after bariatric surgery have not been confirmed. Any anemia that cannot be explained by iron, vitamin B12, or folic acid deficiency should be assessed for possible zinc and copper deficiency. The recommended dosage is 1–2 mg for Cu and 8–15 mg for Zn [22,77,78].

i. Iodine

Iodine is an essential element for the synthesis of thyroid hormones, triiodothyronine and thyroxine, which are necessary for proper development of the fetal nervous system. Mild and moderate iodine deficiency in the mother leads to poorer learning outcomes, neurological impairments, and delayed speech development in the child. Severe iodine deficiency causes congenital iodine deficiency syndrome, stemming from primary hypothyroidism and presenting with severe intellectual impairment [79]. Studies have shown that in iodine sufficient countries, iodine deficiency does not occur after bariatric procedures [80]. However, it needs to be noted that iodine requirements increase during pregnancy due to enhanced thyroid hormone production, increased glomerular filtration of iodine, and placental transfer of iodine [81]. The Royal College of Obstetricians and Gynaecologists recommends iodine supplementation at a dose of 150 µg per day for all pregnant women without a history of thyroid disease. In turn, women with thyroid disorders should have the dose tailored to their current thyroid hormone and thyroid antibody levels [82].

The optimal situation is when vitamin deficiencies are normalized before conception and body weight is already stable. Table 3 provides a summary on supplementation. During pregnancy, complete blood count, folic acid, iron, ferritin, transferrin, vitamin B12, 25-OH-D3, calcium, magnesium, phosphate, PTH, vitamins A and B1, protein, albumin, INR, vitamin K1, and renal and hepatic parameters should be monitored every three months (1×/trimester) [18,83].

## 7. Delivery After Bariatric Surgery

Bariatric surgery itself is not a contraindication to vaginal delivery. A cesarean section is absolutely required only when classical obstetric contraindications are present, e.g., placenta previa, fetal macrosomia, or abnormal fetal position [84]. Available data suggests that prior bariatric surgery may influence delivery outcomes. Women after bariatric procedures have been shown to have lower rates of both elective (18.2% vs. 25%) and emergency cesarean delivery (6.8% vs. 15.1%) compared with BMI-matched controls who have not undergone bariatric surgery before. This reduction may reflect improved metabolic status, lower fetal birth weight, and decreased incidence of labor complications such as shoulder dystocia. Indeed, emergency cesarean delivery due to shoulder dystocia appears less frequent in this population, and the need for instrumental delivery is reduced (5% vs. 6.5%) [85]. It should be noted that the study also demonstrated a reduced risk of postpartum complications, such as hemorrhage, infections, and third- or fourth-degree perineal tears, among women who had undergone bariatric surgery. These findings suggest a potential protective effect of weight loss and metabolic improvement on peripartum outcomes. On the other hand, some studies pointed out that women after bariatric surgery had a higher risk of preterm birth. However, the incidence of preeclampsia, placenta previa, placental abruption, or oligohydramnios was not significantly higher in these women compared with those who had not undergone bariatric surgery [20]. Despite these benefits, women who have undergone bariatric surgery still have higher rates of cesarean delivery and abnormal fetal position compared with women without obesity. Therefore, bariatric surgery does not completely eliminate obstetric risk, and individualized perinatal care remains essential in women with such medical history [86].

The most effective method of relieving labor pain is epidural anesthesia. Even after bariatric surgery, some women will continue to have a BMI above the upper limit of the normal range. In this group, inserting an epidural catheter may be challenging. This is related to the increased difficulty in locating the relevant anatomical landmarks, as well as the thicker subcutaneous tissue. This may increase the risk of procedural failure. In such cases, the use of ultrasound guidance can significantly improve success rates, although it is not yet considered routine practice. Importantly, obesity does not appear to increase the incidence of the most common complication of epidural anesthesia, i.e., post-dural puncture headache, which occurs in this group of patients with a similar or even lower frequency than in women with a normal body weight [87].

## 8. Postpartum Period and Lactation After Bariatric Surgery

During pregnancy and the postpartum period, it is also important to monitor for venous thromboembolism, as obesity is one of its contributing risk factors. This applies especially to patients after a cesarean section who have additional risk factors, including immobilization, thrombophilia, or a previous episode of venous thromboembolism. Weight reduction resulting from bariatric surgery lowers the risk of developing venous thromboembolism. However, if additional risk factors are present despite weight loss, or if alarming symptoms occur, it is recommended to proceed in accordance with the latest guidelines [88]. Venous thromboembolism prevention involves the use of enoxaparin in a prophylactic dose of 40 mg per day (or 40 mg 2×/d in women weighing > 90 kg) in women with confirmed thrombophilia, or in a therapeutic dose of 1 mg/kg of body weight every 12 h in women previously treated with oral anticoagulants following a thromboembolic event, or due to thrombophilia. During pregnancy and breastfeeding, the use of new oral anticoagulants—direct thrombin inhibitors and factor Xa inhibitors—is contraindicated [89].

An issue highlighted in the 2022 review concerns postpartum weight loss [24]. There is still a lack of studies determining which weight loss strategy is most appropriate for women who have undergone bariatric surgery. In women with normal body weight, the most effective approach is a combination of a properly balanced diet and physical activity, provided there are no medical contraindications. Therefore, it seems reasonable to recommend a similar approach for women after bariatric surgery, while taking into account their specific nutritional requirements resulting from the procedure, as well as their individual physical capabilities with regard to physical activity.

There are limited studies addressing the impact of bariatric surgery on breastfeeding. However, given that certain studies report reduced weight gain in infants of mothers who have undergone bariatric surgery, it is considered that supplementation with micro- and macronutrients should be continued during breastfeeding. The same review mentions a study indicating that exclusively breastfed children have lower glucose levels, which may suggest that breastfeeding has a protective effect against childhood obesity [37]. However, when supplementation is provided and the mother has no nutritional deficiencies, the composition of the breastmilk is comparable to that of women who have not undergone surgery. It should be noted that patients after bariatric surgery are at higher risk of reduced breastmilk supply and premature cessation of lactation. Therefore, regular monitoring of the newborn’s weight and support from a lactation consultant is required [89]. Breastfeeding should be continued in the same manner as in every other woman, for at least six months, as recommended by WHO, unless other comorbidities make this inadvisable for the mother or the child.

## 9. Conclusions

Caring for women after bariatric surgery during the periconceptional period and throughout pregnancy requires particular vigilance due to altered gastrointestinal physiology, the risk of macro- and micronutrient deficiencies, and the possibility of atypical pregnancy course. Although bariatric surgery often improves fertility and normalizes menstrual cycles, it is recommended to postpone pregnancy planning for at least 12–18 months to avoid the adverse effects of rapid weight loss. For this reason, careful selection of contraception is recommended, with a preference for methods that do not require intestinal absorption.

Pregnancy after bariatric surgery is associated with a lower risk of certain metabolic complications. However, at the same time, there is an increased likelihood of inadequate maternal weight gain, fetal SGA, and postoperative complications such as hernias or dumping syndrome. Consequently, diagnosing gestational diabetes requires alternative methods that involve monitoring glycaemia rather than using the standard OGTT.

The most common and clinically significant deficiencies involve B vitamins (particularly B12 and B1), iron, folates, vitamins A, D, E, and K, as well as protein. Their occurrence depends on the type of surgery and may lead to serious consequences for both the mother and the fetus. Therefore, regular monitoring of laboratory parameters and appropriately tailored supplementation are essential for the safe management of pregnancy.

Vaginal birth is usually possible, and bariatric surgery itself is not a contraindication to it. In turn, epidural anesthesia may be technically more challenging if obesity persists after the bariatric procedure. Continued supplementation with micro- and macronutrients is recommended after delivery, along with support for breastfeeding, which is safe and beneficial for the child’s development.

Future studies should focus on specific nutritional supplementation strategies during pregnancy and the postpartum period. Further research is needed to evaluate long-term outcomes in women who became pregnant after bariatric surgery and their children. In addition, improving multidisciplinary care may contribute to better outcomes for both mothers and fetuses (Table 4).

## Figures and Tables

**Table 1:** Physiological gestational weight gain based on pre-pregnancy nutritional status.

Pre-Pregnancy Nutritional Status (BMI)	Recommended Total Gestational Weight Gain (from Conception to Delivery)	Recommended Mean Weekly Weight Gain in 2nd and 3rd Trimesters
Normal body weight (18.5–24.9 kg/m^2^)	11.5–16.0 kg	0.42 (0.35–0.5) kg/wk
Overweight (25.0–29.9 kg/m^2^)	7.0–11.5 kg	0.28 (0.23–0.33) kg/wk
Obesity (≥30 kg/m^2^)	5.0–9.0	0.22 (0.17–0.27) kg/wk

**Table 2:** Benefits and risks of pregnancy after bariatric surgery.

Benefits	Risks
Reduced risk of gestational diabetes	Increased risk of small for gestational age (SGA)
Reduced risk of hypertensive disorders/preeclampsia	Increased risk of preterm birth
Reduced risk of perinatal complications	Increased risk of low birth weight
Reduced risk of macrosomia (LGA)	Internal hernia (~5% after gastric bypass)
	Gastric band slippage (~12% during pregnancy)
	Dumping syndrome (40–76% RYGB; up to 40% LSG)

**Table 3:** Suggested supplementation.

Micro- and Macronutrient Deficiencies	Regular Supplementation for Pregnant Women After Bariatric Surgery	Additional Supplementation
Vitamin B12	1 mg of vitamin B12 orally per day or 1000 µg intramuscularly every 12 weeks [22]	
Folic Acid	1 mg per day [18,51]	Alternatively 4 to 5 mg of folic acid per day [52]
Iron	45–60 mg of elemental iron [48,58,59]	
Vitamin A	should not exceed 5000 IU per day [62]	
Vitamin 25-OH-D3	standard 1000 IU per day [22]	In cases of deficiency, 3000–6000 IU per day [22]
Vitamin E	15 mg per day [22,71]	
Vitamin K	10 mg per week [22,34]	
B vitamins	12 mg of thiamine per day [22,37,73]	For women experiencing vomiting: thiamine at 300 mg in a B-complex formulation three times a day, or 100 mg thiamine intramuscularly [22,37,73]
Protein	60–120 g per day, ideally 1.5 g/kg of body weight per day [74,75,76]	Protein intake following BPD needs to be increased by 30% [74,75,76]
Selenium	50 µg per day [22]	
Zinc	8–15 mg per day [22,77,78]	
Copper	1–2 mg per day [22,77,78]	
Iodine	150 µg per day [82]	

**Table 4:** Recommendations’ Summary.

Postpone planning pregnancy after bariatric surgery for 12–18 months (or earlier in exceptional cases if there are no contraindications)
Prefer methods of contraception that do not require intestinal absorption (subdermal implants or levonorgestrel-releasing intrauterine devices)
Maintain adequate gestational weight gain (Table 1)
Diagnose gestational diabetes using alternative methods that involve monitoring glycaemia or HbA1c rather than the standard OGTT
Maintain adequate supplementation (Table 3) during pregnancy and breastfeeding
Monitor every three months (1×/trimester): complete blood count, folic acid, iron, ferritin, transferrin, vitamin B12, 25-OH-D3, calcium, magnesium, phosphate, PTH, vitamins A and B1, protein, albumin, INR, vitamin K1, renal and hepatic parameters
Bariatric surgery itself is not a contraindication to vaginal delivery

## Data Availability

No new data were generated for this study. The study is based on previously published literature, which is cited in the reference list.

## References

[1] Obesity and Overweight. World Health Organization. https://www.who.int/news-room/fact-sheets/detail/obesity-and-overweight.

[2] Davis E.M., Stange K.C., Horwitz R.I. (2012). Childbearing, stress and obesity disparities in women: A public health perspective. Matern. Child Health J..

[3] Janik M.R., Bielecka I., Paśnik K., Kwiatkowski A., Podgórska L. (2015). Female Sexual Function Before and After Bariatric Surgery: A Cross-Sectional Study and Review of Literature. Obes. Surg..

[4] Yang S.T., Liu C.H., Ma S.H., Chang W.H., Chen Y.J., Lee W.L., Wang P.H. (2022). Association between Pre-Pregnancy Overweightness/Obesity and Pregnancy Outcomes in Women with Polycystic Ovary Syndrome: A Systematic Review and Meta-Analysis. Int. J. Environ. Res. Public Health.

[5] Fuchs A., Dulska A., Drosdzol-Cop A. (2020). Is Weight Just a Number? Relationship between Overweight, Obesity and Domains of Sexual Functioning among Young Women. Ginekol. Pol..

[6] Paul R., Drott J., Olbers T., Frisk J., Andersson E. (2023). Motherhood and Motivations for Bariatric Surgery—A Qualitative Study. Hum. Fertil..

[7] Kwong W., Tomlinson G., Feig D.S. (2018). Maternal and Neonatal Outcomes after Bariatric Surgery; a Systematic Review and Meta-Analysis: Do the Benefits Outweigh the Risks?. Am. J. Obstet. Gynecol..

[8] Christopher K.M., Abdelsalam A., Flick L., Xaverius P. (2022). Pregnancy Complications in Women with Weight Loss Surgery Compared to a Non-Surgical Population of Women with Obesity. Obes. Surg..

[9] Mission J.F., Marshall N.E., Caughey A.B. (2015). Pregnancy Risks Associated with Obesity. Obstet. Gynecol. Clin. N. Am..

[10] Blomberg M.I., Källén B. (2010). Maternal Obesity and Morbid Obesity: The Risk for Birth Defects in the Offspring. Birth Defects Res. A Clin. Mol. Teratol..

[11] Hult M., te Riele W., Fischer L., Röstad S., Orava K., Heikkinen T., Sandbu R., Juuti A., Bonn S.E. (2022). Women’s Reasons to Seek Bariatric Surgery and Their Expectations on the Surgery Outcome—A Multicenter Study from Five European Countries. Obes. Surg..

[12] Glazer S., Biertho L. Canadian Adult Obesity Clinical Practice Guidelines: Bariatric Surgery: Selection & Pre-Operative Workup. https://obesitycanada.ca/guidelines/preop.

[13] Brown W.A., Liem R., Al-Sabah S., Anvari M., Boza C., Cohen R.V., Ghaferi A., Våge V., Himpens J., Kow L. (2024). Metabolic Bariatric Surgery Across the IFSO Chapters: Key Insights on the Baseline Patient Demographics, Procedure Types, and Mortality from the Eighth IFSO Global Registry Report. Obes. Surg..

[14] Benhalima K., Minschart C., Ceulemans D., Bogaerts A., Van Der Schueren B., Mathieu C., Devlieger R. (2018). Screening and Management of Gestational Diabetes Mellitus after Bariatric Surgery. Nutrients.

[15] Lee R., Mathew C.J., Jose M.T., Elshaikh A.O., Shah L., Cancarevic I. (2020). A Review of the Impact of Bariatric Surgery in Women with Polycystic Ovary Syndrome. Cureus.

[16] Małczak P., Wysocki M., Dowgiałło-Gornowicz N., Kawa I., Siuda K., Jasińska J., Wójtowicz A., Pisarska-Adamczyk M., Pędziwiatr M., Major P. (2025). Sexual Well-Being After Bariatric Surgery Assessed with New Sexual Satisfaction Scale: A Case-Matched Study of Men and Women. Obes. Surg..

[17] Mechanick J.I., Youdim A., Jones D.B., Timothy Garvey W., Hurley D.L., Molly McMahon M., Heinberg L.J., Kushner R., Adams T.D., Shikora S. (2013). Clinical Practice Guidelines for the Perioperative Nutritional, Metabolic, and Nonsurgical Support of the Bariatric Surgery Patient—2013 Update: Cosponsored by American Association of Clinical Endocrinologists, the Obesity Society, and American Society for Metabolic & Bariatric Surgery. Endocr. Pract..

[18] (2019). ACOG Committee Opinion No. 775 Nonobstetric Surgery during Pregnancy. Obstet. Gynecol..

[19] Bel Lassen P., Tropeano A.I., Arnoux A., Lu E., Romengas L., Katsahian S., Ségrestin B., Lelièvre B., Mitanchez D., Gascoin G. (2025). Maternal and Neonatal Outcomes of Pregnancies after Metabolic Bariatric Surgery: A Retrospective Population-Based Study. Lancet Reg. Health Eur..

[20] Voros C., Varthaliti A., Bananis K., Mavrogianni D., Athanasiou D., Athanasiou A., Athanasiou A., Papahliou A.M., Zografos C.G., Kondili P. (2025). The Relationship Between Obesity, Bariatric Surgery, and Infertility: A Systematic Review. Life.

[21] Shawe J., Ceulemans D., Akhter Z., Neff K., Hart K., Heslehurst N., Štotl I., Agrawal S., Steegers-Theunissen R., Taheri S. (2019). Pregnancy after Bariatric Surgery: Consensus Recommendations for Periconception, Antenatal and Postnatal Care. Obes. Rev..

[22] An Overview of Current Contraceptive Modalities and Historical Perspectives: A Review—GREM—Gynecological and Reproductive Endocrinology & Metabolism. https://gremjournal.com/journal/01-2024/an-overview-of-current-contraceptive-modalities-and-historical-perspectives-a-review/.

[23] Cheah S., Gao Y., Mo S., Rigas G., Fisher O., Chan D.L., Chapman M.G., Talbot M.L. (2022). Fertility, Pregnancy and Post Partum Management after Bariatric Surgery: A Narrative Review. Med. J. Aust..

[24] Schlatter J. (2017). Oral Contraceptives after Bariatric Surgery. Obes. Facts.

[25] Busetto L., Dicker D., Azran C., Batterham R.L., Farpour-Lambert N., Fried M., Hjelmesæth J., Kinzl J., Leitner D.R., Makaronidis J.M. (2017). Practical Recommendations of the Obesity Management Task Force of the European Association for the Study of Obesity for the Post-Bariatric Surgery Medical Management. Obes. Facts.

[26] Snoek K.M., Steegers-Theunissen R.P.M., Hazebroek E.J., Willemsen S.P., Galjaard S., Laven J.S.E., Schoenmakers S. (2021). The Effects of Bariatric Surgery on Periconception Maternal Health: A Systematic Review and Meta-Analysis. Hum. Reprod. Update.

[27] Robinson J.A., Burke A.E. (2013). Obesity and Hormonal Contraceptive Efficacy. Womens Health.

[28] Roumen F.J.M.E. (2007). The Contraceptive Vaginal Ring Compared with the Combined Oral Contraceptive Pill: A Comprehensive Review of Randomized Controlled Trials. Contraception.

[29] Ostrowska L., Lech M., Stefańska E., Jastrzebska-Mierzyńska M., Smarkusz J. (2016). The Use of Contraception for Patients after Bariatric Surgery. Ginekol. Pol..

[30] Zwayne N., Lyman E., Ebersole A., Morse J., Rodríguez S. (2024). Society of Family Planning Committee Statement: Contraception and Body Weight. Contraception.

[31] Rasmussen K.M., Yaktine A.L. (2009). Weight Gain During Pregnancy: Reexamining the Guidelines.

[32] Yu Y., Groth S.W. (2023). Risk Factors of Lower Birth Weight, Small-for-Gestational-Age Infants, and Preterm Birth in Pregnancies Following Bariatric Surgery: A Scoping Review. Arch. Gynecol. Obstet..

[33] Heusschen L., Krabbendam I., van der Velde J.M., Deden L.N., Aarts E.O., Merién A.E.R., Emous M., Bleumink G.S., Lutgers H.L., Hazebroek E.J. (2021). A Matter of Timing-Pregnancy After Bariatric Surgery. Obes. Surg..

[34] Montvignier Monnet A., Savoy D., Préaubert L., Hoffmann P., Bétry C. (2022). In Underweight Women, Insufficient Gestational Weight Gain Is Associated with Adverse Obstetric Outcomes. Nutrients.

[35] Huang X., Liu J., Qi L., Adachi J.D., Wu J., Li Z., Meng Q., Li G., Lip G.Y.H. (2022). Birth Weight and the Risk of Cardiovascular Outcomes: A Report From the Large Population-Based UK Biobank Cohort Study. Front. Cardiovasc. Med..

[36] Alamri S.H., Abdeen G.N. (2022). Maternal Nutritional Status and Pregnancy Outcomes Post-Bariatric Surgery. Obes. Surg..

[37] Khambalia A.Z., Algert C.S., Bowen J.R., Collie R.J., Roberts C.L. (2017). Long-Term Outcomes for Large for Gestational Age Infants Born at Term. J. Paediatr. Child. Health.

[38] Derraik J.G.B., Maessen S.E., Gibbins J.D., Cutfield W.S., Lundgren M., Ahlsson F. (2020). Large-for-Gestational-Age Phenotypes and Obesity Risk in Adulthood: A Study of 195,936 Women. Sci. Rep..

[39] Al-Nimr R.I., Hakeem R., Moreschi J.M., Gallo S., McDermid J.M., Pari-Keener M., Stahnke B., Papoutsakis C., Handu D., Cheng F.W. (2019). Effects of Bariatric Surgery on Maternal and Infant Outcomes of Pregnancy—An Evidence Analysis Center Systematic Review. J. Acad. Nutr. Diet..

[40] Gonzalez-Urquijo M., Zambrano-Lara M., Patiño-Gallegos J.A., Rodarte-Shade M., Leyva-Alvizo A., Rojas-Mendez J. (2021). Pregnant Patients with Internal Hernia after Gastric Bypass: A Single-Center Experience. Surg. Obes. Relat. Dis..

[41] Morgan H.D., Morrison A.E., Hamza M., Jones C., Cassar C.B., Meek C.L. (2024). The Approach to a Pregnancy after Bariatric Surgery. Clin. Med..

[42] Kahramangil B., Menzo E.L., Szomstein S., Rosenthal R. (2023). Dumping Syndrome. The SAGES Manual of Physiologic Evaluation of Foregut Diseases.

[43] Rodrigues-Martins D., Nunes I., Monteiro M.P. (2025). The Challenges of Diagnosing Gestational Diabetes after Bariatric Surgery: Where Do We Stand?. Obes. Facts.

[44] Krzizek E.C., Brix J.M., Stöckl A., Parzer V., Ludvik B. (2021). Prevalence of Micronutrient Deficiency after Bariatric Surgery. Obes. Facts.

[45] Gernand A.D., Schulze K.J., Stewart C.P., West K.P., Christian P. (2016). Micronutrient Deficiencies in Pregnancy Worldwide: Health Effects and Prevention. Nat. Rev. Endocrinol..

[46] Nunes R., Santos-Sousa H., Vieira S., Nogueiro J., Bouça-Machado R., Pereira A., Carneiro S., Costa-Pinho A., Lima-da-Costa E., Preto J. (2022). Vitamin B Complex Deficiency After Roux-En-Y Gastric Bypass and Sleeve Gastrectomy—A Systematic Review and Meta-Analysis. Obes. Surg..

[47] Shipton M.J., Johal N.J., Dutta N., Slater C., Iqbal Z., Ahmed B., Ammori B.J., Senapati S., Akhtar K., Summers L.K.M. (2021). Haemoglobin and Hematinic Status Before and After Bariatric Surgery over 4 Years of Follow-Up. Obes. Surg..

[48] Dong J., Yin L.L., Deng X.D., Ji C.Y., Pan Q., Yang Z., Peng T., Wu J.N., Wu G.H., Zhou L.Y. (2023). Initiation and Duration of Folic Acid Supplementation in Preventing Congenital Malformations. BMC Med..

[49] Vynckier A.K., Ceulemans D., Vanheule G., De Mulder P., Van Den Driessche M., Devlieger R. (2021). Periconceptional Folate Supplementation in Women after Bariatric Surgery—A Narrative Review. Nutrients.

[50] O’Kane M., Parretti H.M., Pinkney J., Welbourn R., Hughes C.A., Mok J., Walker N., Thomas D., Devin J., Coulman K.D. (2020). British Obesity and Metabolic Surgery Society Guidelines on Perioperative and Postoperative Biochemical Monitoring and Micronutrient Replacement for Patients Undergoing Bariatric Surgery—2020 Update. Obes. Rev..

[51] National Institute for Health and Care Excellence (NICE) Maternal and Child Nutrition: Nutrition and Weight Management in Pregnancy, and Nutrition in Children up to 5 Years. https://www.nice.org.uk/guidance/ng247.

[52] Sandvik J., Bjerkan K.K., Græslie H., Hoff D.A.L., Johnsen G., Klöckner C., Mårvik R., Nymo S., Hyldmo Å.A., Kulseng B.E. (2021). Iron Deficiency and Anemia 10 Years After Roux-En-Y Gastric Bypass for Severe Obesity. Front. Endocrinol..

[53] Steenackers N., Van Der Schueren B., Mertens A., Lannoo M., Grauwet T., Augustijns P., Matthys C. (2018). Iron Deficiency after Bariatric Surgery: What Is the Real Problem?. Proc. Nutr. Soc..

[54] Ruz M., Carrasco F., Rojas P., Codoceo J., Inostroza J., Basfi-fer K., Valencia A., Csendes A., Papapietro K., Pizarro F. (2012). Heme- and Nonheme-Iron Absorption and Iron Status 12 Mo after Sleeve Gastrectomy and Roux-En-Y Gastric Bypass in Morbidly Obese Women. Am. J. Clin. Nutr..

[55] Ubom A.E., Begum F., Ramasauskaite D., Nieto-Calvache A.J., Oguttu M., Nunes I., Malel Z.J., Beyeza-Kashesya J., Wright A. (2025). FIGO Good Practice Recommendations on Anemia in Pregnancy, to Reduce the Incidence and Impact of Postpartum Hemorrhage (PPH). Int. J. Gynecol. Obstet..

[56] Abu-Ouf N.M., Jan M.M. (2015). The Impact of Maternal Iron Deficiency and Iron Deficiency Anemia on Child’s Health. Saudi Med. J..

[57] Parrott J., Frank L., Rabena R., Craggs-Dino L., Isom K.A., Greiman L. (2017). American Society for Metabolic and Bariatric Surgery Integrated Health Nutritional Guidelines for the Surgical Weight Loss Patient 2016 Update: Micronutrients. Surg. Obes. Relat. Dis..

[58] Huang B., Yo J.H., Gandhi S., Maxwell C. (2021). Micronutrient Screening, Monitoring, and Supplementation in Pregnancy after Bariatric Surgery. Obstet. Med..

[59] Heusschen L., Berendsen A.A.M., van Bon A.C., van Laar J.O.E.H., Krabbendam I., Hazebroek E.J. (2024). Nutrient Status and Supplement Use During Pregnancy Following Metabolic Bariatric Surgery: A Multicenter Observational Cohort Study. Obes. Surg..

[60] Slater C., Morris L., Ellison J., Syed A.A. (2017). Nutrition in Pregnancy Following Bariatric Surgery. Nutrients.

[61] Maia S.B., Souza A.S.R., Caminha M.D.F.C., da Silva S.L., Cruz R.d.S.B.L.C., Dos Santos C.C., Filho M.B. (2019). Vitamin A and Pregnancy: A Narrative Review. Nutrients.

[62] Kaska L., Kobiela J., Abacjew-Chmylko A., Chmylko L., Wojanowska-Pindel M., Kobiela P., Walerzak A., Makarewicz W., Proczko-Markuszewska M., Stefaniak T. (2013). Nutrition and Pregnancy after Bariatric Surgery. ISRN Obes..

[63] Wei J.H., Lee W.J., Chong K., Lee Y.C., Chen S.C., Huang P.H., Lin S.J. (2018). High Incidence of Secondary Hyperparathyroidism in Bariatric Patients: Comparing Different Procedures. Obes. Surg..

[64] Wang S., Shi M., Zhou L., Huang H., Mo S. (2023). Correlation of Vitamin E Level during Pregnancy with Maternal and Neonatal Health Outcomes: A Meta-Analysis and Systematic Review. Am. J. Transl. Res..

[65] Shi H., Jiang Y., Yuan P., Chen L., Gong X., Yang Y., Wang Y., Jiang H., Li Y., Sun M. (2022). Association of Gestational Vitamin E Status with Pre-Eclampsia: A Retrospective, Multicenter Cohort Study. Front. Nutr..

[66] Iswara R.P., Yunus K., Pati N. (2023). Vitamin K Deficiency. Causes and Management of Nutritional Deficiency Disorders.

[67] Lammers S., Iovino N.A., Pusateri A., Snyder A., Butnariu M., Frey H.A., Skeans J. (2025). Maternal and Neonatal Hemorrhage From Vitamin K Deficiency in the Setting of Crohn Disease in Pregnancy. Obstet. Gynecol..

[68] Nijsten K., Van Der Minnen L., Wiegers H.M.G., Koot M.H., Middeldorp S., Roseboom T.J., Grooten I.J., Painter R.C. (2022). Hyperemesis Gravidarum and Vitamin K Deficiency: A Systematic Review. Br. J. Nutr..

[69] Lee S., Kim H.M., Kang J., Seong W.J., Kim M.J. (2022). Fetal Intracranial Hemorrhage and Maternal Vitamin K Deficiency Induced by Total Parenteral Nutrition: A Case Report. Medicine.

[70] Jans G., Guelinckx I., Voets W., Galjaard S., Van Haard P.M.M., Vansant G.M., Devlieger R. (2014). Vitamin K1 Monitoring in Pregnancies after Bariatric Surgery: A Prospective Cohort Study. Surg. Obes. Relat. Dis..

[71] Mihatsch W.A., Braegger C., Bronsky J., Campoy C., Domellöf M., Fewtrell M., Mis N.F., Hojsak I., Hulst J., Indrio F. (2016). Prevention of Vitamin K Deficiency Bleeding in Newborn Infants: A Position Paper by the ESPGHAN Committee on Nutrition. J. Pediatr. Gastroenterol. Nutr..

[72] Devlieger R., Guelinckx I., Jans G., Voets W., Vanholsbeke C., Vansant G. (2014). Micronutrient Levels and Supplement Intake in Pregnancy after Bariatric Surgery: A Prospective Cohort Study. PLoS ONE.

[73] Ganipisetti V.M., Naha S. (2023). Bariatric Surgery Malnutrition Complications. StatPearls.

[74] Kamal F.A., Fernet L.Y., Rodriguez M., Kamal F., Da Silva N.K., Kamal O.A., Aguilar A.A., Arruarana V.S., Ramirez M.M., Kamal F.A. (2024). Nutritional Deficiencies Before and After Bariatric Surgery in Low- and High-Income Countries: Prevention and Treatment. Cureus.

[75] Lupoli R., Lembo E., Saldalamacchia G., Avola C.K., Angrisani L., Capaldo B. (2017). Bariatric Surgery and Long-Term Nutritional Issues. World J. Diabetes.

[76] O’Kane M., Barth J.H. (2016). Nutritional Follow-up of Patients after Obesity Surgery: Best Practice. Clin. Endocrinol..

[77] Różańska-Walędziak A., Walędziak M., Mierzejewska A., Skopińska E., Jędrysik M., Chełstowska B. (2023). Nutritional Implications of Bariatric Surgery on Pregnancy Management-A Narrative Review of the Literature. Medicina.

[78] Ma Z.F., Brough L. (2025). Effect of Iodine Nutrition During Pregnancy and Lactation on Child Cognitive Outcomes: A Review. Nutrients.

[79] Manousou S., Carlsson L.M.S., Eggertsen R., Hulthén L., Jacobson P., Landin-Wilhelmsen K., Trimpou P., Svensson P.A., Nyström H.F. (2017). Iodine Status After Bariatric Surgery—A Prospective 10-Year Report from the Swedish Obese Subjects (SOS) Study. Obes. Surg..

[80] Moreno-Reyes R., Fuentes Peña C., Nuñez J.F., Sánchez M.B., Carvajal J.J., Roble K., Mendoza-León M.J., Rangel-Ramírez M.A., Opazo M.C., Lay M.K. (2025). Critical Role of Iodine and Thyroid Hormones During Pregnancy. Int. J. Mol. Sci..

[81] Chan S.Y., Marsh M.S., Gilbert J., Boelaert K., Evans C., Dhillon-Smith R. (2025). Management of Thyroid Disorders in Pregnancy. BJOG.

[82] Burlina S., Dalfrà M.G., Lapolla A. (2023). Pregnancy after Bariatric Surgery: Nutrition Recommendations and Glucose Homeostasis: A Point of View on Unresolved Questions. Nutrients.

[83] Pg Baharuddin D.M., Payus A.O., Abdel Malek Fahmy E.H., Sawatan W., Than W.W., Abdelhafez M.M., Oo Leik N.K., Ag Daud D.M., Mohd Daud M.N., Ahmad Z.N.S. (2021). Bariatric Surgery and Its Impact on Fertility, Pregnancy and Its Outcome: A Narrative Review. Ann. Med. Surg..

[84] Stephansson O., Johansson K., Söderling J., Näslund I., Neovius M. (2018). Delivery Outcomes in Term Births after Bariatric Surgery: Population-Based Matched Cohort Study. PLoS Med..

[85] Pinnam B.S.M., Ojemolon P.E., Fatima S., Abougergi M.S., Popov V. (2025). Impact of Prior Bariatric Surgery on Labor and Delivery-Related Outcomes: A Nationwide Study. Obes. Surg..

[86] Liu Z., Zhu J. (2024). Advances in Epidural Labor Analgesia for Obese Parturients. J. Pain Res..

[87] Navti O.B., Pavord S. (2024). Venous Thromboembolism in Pregnant Obese Individuals. Best. Pract. Res. Clin. Obstet. Gynaecol..

[88] Tomkowski W., Kuca P., Urbanek T., Chmielewski D., Krasiński Z., Pruszczyk P., Windyga J., Oszkinis G., Jawień A., Burakowski J. (2017). Venous Thromboembolism—Recommendations on the Prevention, Diagnostic Approach and Management. The 2017 Polish Consensus Statement. Acta Angiol..

[89] Suri P., Bellini A., Bloemhard M.E., Choi J.Y., Hoyt-Austin A., McCreary R.J., Kennedy C., Clapp B., Husain F., Ma P. (2024). Breastfeeding in metabolic and bariatric patients: A comprehensive guide for surgeons, patients, and the multidisciplinary team. Surg. Obes. Relat. Dis..

